# Temperature and pH Stability of Anthraquinones from Native *Aloe vera* Gel, Spray-Dried and Freeze-Dried *Aloe vera* Powders during Storage

**DOI:** 10.3390/foods11111613

**Published:** 2022-05-30

**Authors:** Uzma Sadiq, Harsharn Gill, Jayani Chandrapala

**Affiliations:** School of Science, RMIT University, Bundoora, Melbourne, VIC 3083, Australia; uzma.sadiq@student.rmit.edu.au (U.S.); harsharn.gill@rmit.edu.au (H.G.)

**Keywords:** *Aloe vera*, anthraquinones, stability, aloin, aloe-emodin, rhein, spray drying, freeze drying, processing temperature, pH

## Abstract

The present study explored the stability of extracted anthraquinones (aloin, aloe-emodin and rhein) from whole-leaf *Aloe vera* gel (WLAG), its freeze-dried powder (FDP) and spray-dried powder (SDP) under varying pH and temperature conditions during storage. Each anthraquinone behaved differently under different processing parameters. The amount of anthraquinones present in the gel was higher than in FDP and SDP. The aloin contents decreased by more than 50% at 50 °C and 70 °C, while at 25 °C and 4 °C, the decrease was moderate. A substantial reduction in aloin concentration was noticed at pH 6.7, whereas it remained unaffected at pH 3.5. The temperature and pH had no significant effect on the stability of aloe-emodin. Interestingly, a small quantity of rhein was detected during storage due to the oxidative degradation of aloin into aloe-emodin and rhein. These findings can provide significant insight into retaining anthraquinones during processing while developing functional foods and nutraceuticals to obtain maximum health benefits.

## 1. Introduction

*Aloe vera* (*Aloe barbadensis*) is a perennial, drought-resistant succulent plant that belongs to the Liliaceae family. Out of 360 known species of Aloe vera, *Aloe arborescens* Miller; *A. perryi* Baker; *A. ferox* Miller o’ *A. capensis*; and *Aloe bar-badensis* Miller, also known as *Aloe vera* Linne o’ *A. vulgaris* Lamarck have medicinal properties [1,2]. The Aloe vera juicy material can be divided into two parts: the yellow exudate which contains a high percentage of aloin and other anthraquinones (such as aloe-emodin, barbaloin isobarbaloin, emodin, anthracene, anthranol) and the gel which contains 99.5% water along with polysaccharides, vitamins, enzymes, amino acids, phenolic compounds, phytosterols, and anthraquinones [3,4,5].

Anthraquinone derivatives, including aloin (A and B), barbaloin, isobarbaloin, emodin, aloe-emodin, rhein, chrysophanol and physcion, are known to possess a wide range of biological activities. These include laxative, antibacterial, antiviral [6], anti-inflammatory, antifungal, anticancer, diuretic, hepatoprotective, vasorelaxant and antioxidant effects [7,8,9,10,11,12,13]. Kang et al. [14] demonstrated the inhibitory activities of anthraquinones against advanced glycation end-products (AGEs) that are formed during food processing, and Huang et al. [15] reported enhanced anticancer activity of low-dose cisplatin when used in combination with anthraquinones against human gastric cancer cells in vitro.

Despite numerous studies reporting the beneficial effects of anthraquinones, their use in the food industry has been limited because of their poor water solubility, instability and rapid degradability during secondary processing conditions [16,17]. The five free anthraquinones (chrysophanol, emodin, physcion, aloe-emodin and rhein) extracted from rhubarb showed different thermal properties [18]. It was also found that aloe-emodin and emodin are highly prone to acidic and hydrolytic degradation, moderately prone to oxidative and thermal degradation (105 °C) and least prone to basic degradation [19]. Anthraquinone concentrations vary greatly depending upon climatic conditions, species, and even within various parts of the same plant [11,20,21]. Chang and colleagues [22] reported instability of polysaccharides and barbaloin upon heating and in various solvents, respectively, though exact mechanisms of hydrolysis remain unclear. However, Pellizzoni et al. [23] further reported that this instability could not be improved by the addition of ascorbates as well as antimicrobial agents. Ding et al. [16] investigated the stability of pure aloin in phosphate buffers by differing pH, temperature and light, and reported its decomposition at 70 °C and pH 8.0. Machado and colleagues developed and validated an HPLC method to detect aloin in fresh and dried leaves of *Aloe vera* by using phosphate-buffered saline (pH 3) as an extraction solvent [21].

The use of fresh *Aloe vera* gel in various processed foods is unsustainable due to its perishability and quick ability to relinquish its functional properties. However, various drying technologies that can dramatically improve the storage stability of *Aloe vera* gel have been explored to enhance its widespread use in food products. Spray drying is the most often employed method, followed by freeze drying, although the latter is a mild process to produce a powder. Dehydrated *Aloe vera* samples produced by spray drying, industrial freeze drying, reactance window drying and radiant zone drying led to lower water activities, higher solubilities, and showed high hygroscopic behaviours compared to fresh *Aloe vera* gels [24].

Other plants, especially *Rheum, Rumex*, *Rhamnus* and *Cassia*, contain anthraquinones that are sensitive to various processing conditions. McDougall et al. [25] evaluated the effect of various cooking methods on *Rheum rhapontigen* and found a dramatic reduction in anthraquinone content (aglycones and glycones) within 10 min of baking. However, Yen and Chung [26] reported the thermal degradation of anthraquinones extracted from *Cassia tora* seeds during roasting.

To date, no study has examined the effect of storage temperature and pH on the stability of anthraquinones extracted from *Aloe vera* leaves. The stability of anthraquinones in spray-dried and freeze-dried *Aloe vera* powders also remains unknown. Therefore, the present work was conducted to examine the stability of three anthraquinone compounds (aloin, aloe-emodin, rhein) present in fresh and stored whole-leaf *Aloe vera* gel, spray-dried powder and freeze-dried powder under various temperature and pH conditions. FTIR spectroscopy was also used to analyse the effect of heat, pH and drying procedures on the structure of anthraquinones.

## 2. Materials and Methods

### 2.1. Materials

*Aloe vera* (*Aloe barbadensis*) leaves were obtained from Aloe vera Australia (Goodman international, Brisbane, Australia). Standards of aloin (Mikromol, item code: MM1318.01, CAS# 1415-73-2), emodin (LGC, item code: DRE-C13117900, CAS #518-82-1), aloe-emodin (TRC, item code: A575400-10MG, CAS #481-72-1), and rhein CRS (EDQM, item code: EPY0002159, CAS# 478-43-3) were purchased from Novachem Pty Ltd. (Melbourne, Australia). HPLC-grade methanol and formic acid and analytical-grade HCL and NaOH were purchased from Sigma Aldrich Pty Ltd. (Castle Hill, NSW, Australia). MilliQ water was used at all times.

### 2.2. Preparation of Whole-Leaf Aloe vera Gel

*Aloe vera* leaves along with rinds were washed with MilliQ water and cut into small pieces. These cut slices were blended using an electric blender (Nutri Ninja) to prepare the mucilaginous gel, followed by centrifugation and vacuum filtration to eliminate the rind portions. The liquid gel was then stored at −20 °C overnight until further analysis.

### 2.3. Preparation of Whole-Leaf Aloe vera Spray-Dried and Freeze-Dried Powders

Spray-dried whole-leaf *Aloe vera* powder was prepared using a mini spray dryer B-290 (BÜCHI Labor Technik AG, Meierseggstrasse 40 Postfach, Flawil, Switzerland) which operated at the inlet and outlet temperatures of 175 °C and 75 °C, respectively.

Freeze-dried whole-leaf *Aloe vera* powder was prepared by pre-freezing the whole-leaf *Aloe vera* gel (WLAG) at −80 °C for 24 h and then lyophilising in a freeze dryer (VirTis Model EL-SP Scientific, Hardning Highway, Buena, NJ), USA) for ten days at temperature and pressures of −40 °C and 0.25 atm, respectively.

### 2.4. Evaluation for Temperature and pH Stability

The temperature and pH stability of anthraquinones in whole-leaf *Aloe Vera* gel (WLAG), spray-dried whole-leaf *Aloe vera* powder (SDP) and freeze-dried whole-leaf *Aloe vera* powder (FDP) were studied at four different temperatures (4 °C, 25 °C, 50 °C and 70 °C) and five different pH values (3.5, 4.6, 5.6, 6.4, 6.7) at room temperature.

An accurately weighed sample portion (10 g each) was transferred into four test tubes to test the stability towards temperature. One tube was left in the refrigerator at 4 °C, while another tube was left at room temperature (25 °C). The other two tubes with WLAG were heated at 50 °C and 70 °C by using a water bath TW-20 (JULABO, Marcon Boulevard, Allentown, PA, USA) for 15 min. The same procedure was also repeated for 3- and 7-day samples.

For testing the pH stability, the sample pH was adjusted by adding 1M HCL or 1M NaOH, using the pH SevenCompact^TM^ S220 (Mettler-Toledo GmbH, Im Langacher, Greifensee, Switzerland). HPLC analysis was performed on days 0, 3 and 7. All tubes were stored at room temperature and covered with aluminium foil to protect them from light.

### 2.5. Quantification of Anthraquinones by HPLC

The WLAG, pH-adjusted WLAGs and heat-treated (4 °C, 25 °C, 50 °C and 70 °C) WLAGs, SDP and FDP, were centrifuged at 6000 rpm for 20 min (Allegra 64R-Beckman Coulter, NSW, Australia). The supernatant was collected, and ten millilitre aliquots of methanol were added and vortex mixed, followed by sonication for 10 min using an ultrasonic bath (FXP12, Unisonics, NSW, Australia) at ~4 ± 1 °C. The supernatant was taken carefully and filtered by a 0.45 µm membrane filter for all samples prior to HPLC analysis.

Stock solutions (1000 µg/mL) of aloin, aloe-emodin and rhein were prepared by dissolving in HPLC grade methanol and used as standards. Working solutions of mixed standards were prepared at concentrations of 5 ppm, 10 ppm, 20 ppm and 40 ppm by diluting the stock solution in a volumetric flask with methanol. Both stock and working solutions were prepared on the day of analysis and injected directly into the HPLC system. HPLC measurements were performed on an Agilent series1250 infinity gradient HPLC (Agilent Technologies, Santa Clara, CA, USA) equipped with a 600 solvent pump and a C_18_ reversed-phase packing column (Phenomenex XB-C18, 250 mm × 4.60 mm, 3.6 μm Aeris). Gradient elution was performed at 0.7 mL/min, usinga binary mobile phase consisting of water with 1% formic acid (A) and methanol (B). The elution was monitored at 254 nm and the injection volume was 20 μL. The HPLC run started with 20% methanol and increased to 30%, 45% and 60% at 3, 10 and 15 min, respectively. The methanol percentage was increased to 70% at 18 min and maintained for 8 min, followed by a gradual decrease to 60%, 40% and 20% at 30, 32 and 35 min, respectively.

A calibration curve was established for each standard as a function of their peak areas. The contents of anthraquinones present in the samples were quantified against the standards. The amount of anthraquinones was presented as micrograms per gram of dry powder in the case of spray-dried and freeze-dried powders of *Aloe vera*, while for fresh whole-leaf *Aloe vera* gel, it was the microgram per gram of solid, obtained after drying a measured quantity of fresh whole-leaf *Aloe vera* gel (WLAG) in the oven at 102 °C until a constant weight was obtained.

### 2.6. Fourier Transform Infrared (FTIR) Spectroscopy Analysis

FTIR spectra of extracted anthraquinones from all samples were obtained using the FTIR (Spectrum two, Perkin Elmer, Australia) furnished with IRWinLab FTIR software. Measurements were taken at the wavelength range of 4000–400 cm^−1^ using Milli Q water as the background. Sixteen scans were performed, and the resolution of 4 cm^−1^ was used.

### 2.7. Statistical Analysis

All measurements were performed in triplicates, and the results were expressed in means ± standard deviation. Statistical differences for quantification of anthraquinones in the controls (Table 1) were analysed by one-way analysis of variance (ANOVA) using Tukey’s post hoc HSD test to establish a significant difference between samples studied (WLAG, SDP and FDP) with 95% confidence interval by SPSS 11.0 software (SPSS Inc., Chicago, IL, USA). For performing temperature and pH stability comparisons compared to the controls, the data were analysed using a two-way analysis of variance (ANOVA) followed by Dunnett’s multiple comparisons. Values significantly different from controls to various temperature and pH treatments in a two-way ANOVA with Dunnett’s test are indicated with asterisks (* *p* < 0.01, *** *p* < 0.0003, **** *p* < 0.0001). Data analysis was performed using Graph pad Prism 9.1.0 (GraphPad Software, Inc., San Diego, CA, USA). Here, *p*-values of less than 0.05 were considered significant for all statistical tests.

## 3. Results and Discussion

### 3.1. Quantification of Anthraquinones through HPLC

**Figure 1:**
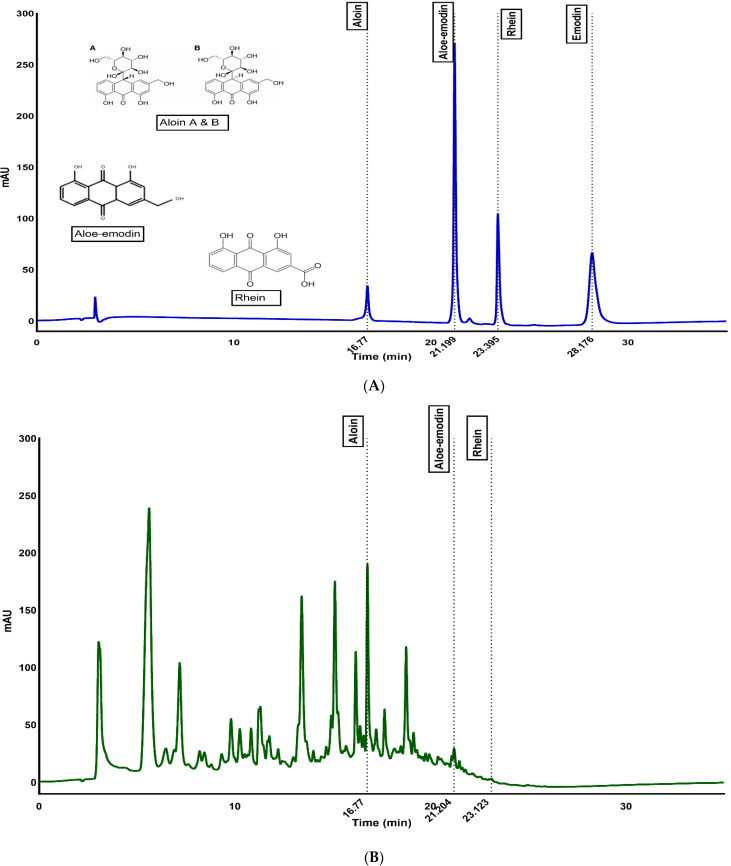
HPLC chromatographs of (**A**) reference standards of aloin, aloe-emodin and rhein and (**B**) WLAG extract containing aloin, aloe-emodin and rhein.

Figure 1A shows the baseline separation of anthraquinones in 35 min with retention times of 16.74, 21.21, 23.40 and 28.26 for aloin, aloe-emodin, rhein, and emodin, respectively. Figure 1B is a representative chromatogram of the whole-leaf *Aloe vera* gel sample and shows aloin, aloe-emodin and rhein compounds with the same retention times as standards. The aloin and aloe-emodin contents of whole-leaf *Aloe vera* gel (WLAG on day zero) were significantly different from those of the spray-dried (SDP) and freeze-dried (FDP) powders of *Aloe vera* (Table 1).

The freshly prepared WLAG had the highest amount of aloin with an average of ~6134 µg/g and this were consistent with the concentrations (4040 to 13,410 µg/g of aloin in fresh gel and 96,270 to 247,000 µg/g of aloin in fresh latex) reported by Sánchez-Machado et al. [21]. Some of these differences were due to the usage of different extraction solvents (methanol), as Sánchez-Machado et al. used phosphate-buffered saline of pH 3 to extract aloin (lower pH conserves aloin). In addition, this variability might be due to the growing conditions of *Aloe Vera* as well [27]. Aloin contents in freeze-dried powders were ~223 µg/g, which is lower than previously reported (1140 µg/g of aloin in freeze-dried ethanol extracts; [28]). The lower concentration of aloin in freeze-dried powders might be due to its failure to preserve phenolic glycoside, as previously reported [29]. It might be due to the failure of the methanol solvent to extract aloin efficiently from the freeze-dried powders.

Aloin contents in spray-dried powder were ~220 µg/g, and these were lower in concentration in comparison to the freeze-dried powders because of the exposure of polyphenols to high temperature during spray drying. Although there are no previous data available on the quantification of aloin in spray-dried powders of *Aloe vera*, the values from the present study fall between the observed ranges (150 to 450 µg/g) of aloin in *Aloe vera* leaf powders prepared using sun-drying followed by oven drying (50 °C) methods [11]. Overall, dehydration-process-induced stresses were the reason for low aloin contents in both (FDP and SDP) powders [30]. During spray drying, thermal, automisation, mechanical and dehydration stresses caused the degradation of bioactive components. However, during lyophilisation, dehydration, pH changes, crystallisation and interfacial stresses take place [31]. Moreover, the higher percentages of aloin in WLAG compared to FDP and SDP are due to the size of the solvent (methanol) molecules and the diffusion mechanism through which it can penetrate the solid matrix and increase the polarity of the solvent [32].

The amount of aloe-emodin in WLAG on day zero was ~69 µg/g. This value was one-third of the values reported previously (270 µg/g; [11]). This may be due to the differences in climatic regions from where the products were sourced. *Aloe vera* from warmer climatic regions is reported to have a lower amount of aloe-emodin [11]. Aloe-emodin was not detected in the freeze-dried powders, although this is against the extracted 5.52 ± 0.3 µg/g of aloe-emodin from freeze-dried *Aloe vera* leaves previously reported by Lee et al. [33]. This might be due to the inefficient extraction of aglycones in methanol. Spray-dried powder contained ~18 µg/g of aloe-emodin on day zero of analysis. In the case of spray-dried powders, heating may have liberated the aglycones (aloe-emodin) from their glycosides (aloin). Similar results have previously been reported by Fu [34].

In addition, large variability in the amount of aloin and aloe-emodin was observed in WLAG on day zero (Table 1). A higher quantity of aloin of ~6134.0 µg/g was found compared to aloe-emodin at ~69.0 µg/g, and these findings agree with previous studies [11,35]. The cause for the lesser quantity of aloe-emodin was that it could not be synthesised directly in plants but instead resulted from the oxidative decomposition of its glycosides [36,37].

### 3.2. FTIR Analysis

**Figure 2:**
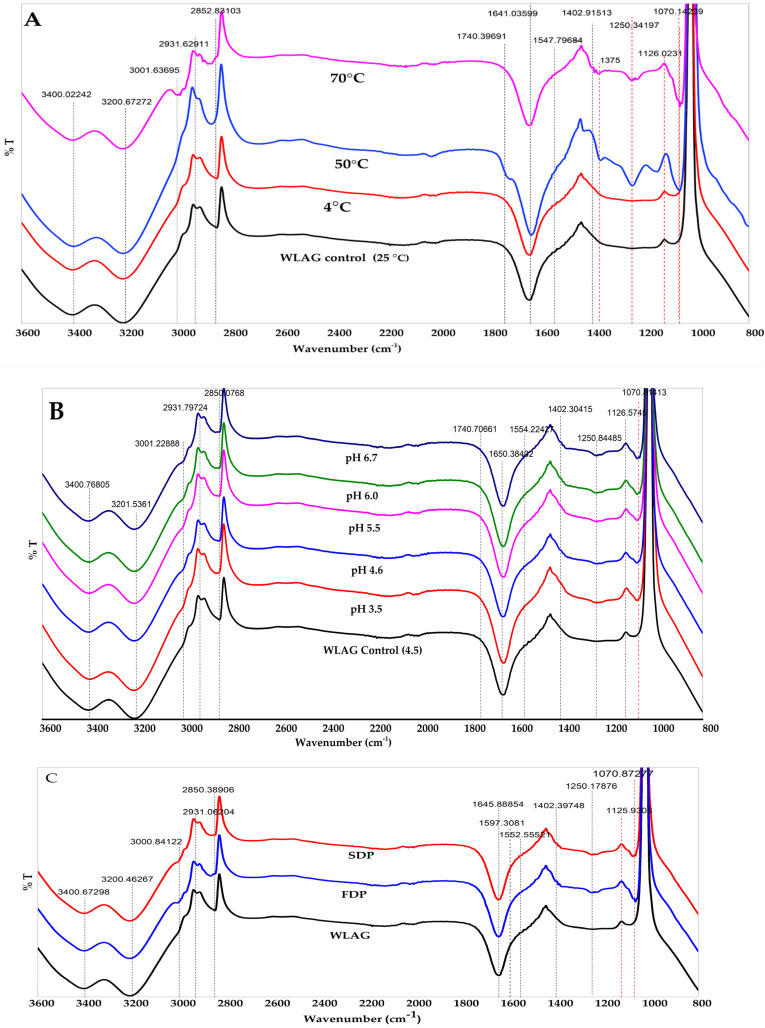
FTIR spectra of (**A**) anthraquinone extracted from whole leaf *Aloe vera* gel (WLAG) at various temperatures, (**B**) anthraquinone extracted from whole leaf *Aloe vera* gel at various pH conditions and (**C**) anthraquinone extracted from the whole leaf *Aloe vera* gel (WLAG), freeze-dried powder (FDP) and spray-dried powder (SDP) of *Aloe vera*.

FTIR profiles of anthraquinones extracted from WLAG exposed to different temperatures (4 °C, 25 °C, 50 °C, 70 °C) and pHs (3.5, 4.6, 5.5, 6.0, 6.7), freeze-dried powders (FDP) and spray-dried powders (SDP) of *Aloe vera* are shown in Figure 2A–C. Anthraquinones are assembled from an anthracene ring through a keto group located at 9,10 carbon as a basic core with diverse functional groups, such as -OH, -CH_3_-COOH, CHO, CH_2_OH and OCH_3_. Figure 1A illustrates the structure of aloin, aloe-emodin, and rhein present in *Aloe vera* plants. In general, all samples exhibited a broad and intense absorption band at 3400 cm^−1^ which can be attributed to the -OH stretching of mannose and uronic acids and bands at 3200 cm^−1^ assigned to O-H stretching of anthraquinones (phenolic groups) such as aloin and aloe-emodin. These compounds contain hydroxyl groups directly attached to an aromatic hydrocarbon. Two bands at 2930 cm^−1^ and 2850 cm^−1^ are related to C-H symmetric stretching and asymmetric stretching of methylene (CH_2_), respectively, indicating long aliphatic chains (-CH) of anthraquinones. Peaks at 1725 cm^−1^ of (C=O) stretching suggest the presence of carbonyl groups. Aromatic double bonds (C=C) of benzene rings could be seen at 1546 cm^−1^ and 1641 cm^−1^, indicating vinyl ether and the aloin compounds.

The minor peaks at 1402 cm^−1^ are from the -COO of carboxylate compounds. Peaks between 1250 cm^−1^ and 1000 are the C-O-C stretching of (COCH_3_), probably related to methyl acyl groups, indicating the presence of *O*-acetyl ester. Peaks at 1070 cm^-1^ and 1126 cm^−1^ are associated with the C–O and C–H bending vibration associated with rhamnogalacturonan, a side-chain constituent of pectins [38,39,40,41].

The peak patterns obtained from FTIR (Figure 2A) for samples heated at 50 °C and 70 °C indicated a change in the chemical structure of aloin at wavenumbers 1740 cm^−1^ and 1250 cm^−1^ corresponding to the C = O and C-O-C stretches of carbonyl groups of *O*-acetyl ester. The peak labelled 50 °C (Figure 2A) has two extra sharp edges at 1250 cm^−1^ and 1740 cm^−1^, and in the case of 70 °C, these are not prominent. WLAG samples at 25 °C and at 4 °C) showed no peaks at 1250 cm^−1^ and 1740 cm^−1^, indicating the absence of any structural modifications. However, in Figure 2A, strong peaks were identified at 1070 cm^−1^ and 1126 cm^−1^ in heated WLAG (50 °C and 70 °C) which correspond to C-O and C-H bonds as a result of glycosidic breakage and release of sugars (mannose or glucose) in aloin, as previously reported by Nejatzadeh-Barandozi and Enferadi and Patel et al. [42,43]. The high-intensity peaks at 1402 cm^−1^ are assigned to symmetrical -COO stretching of carboxylate due to de-esterification exaggerated after heating [41]. This was previously reported by Lim and Cheong, who analysed the de-esterification of pectin by mixing pure *Aloe vera* gel with ethanol and heating it at various temperatures [39]. A change in peak intensity at 1641 cm^−1^ in samples heated at 50 °C and 70 °C relates to the hydrolysis of aloin by an enzyme polygalacturonase (PG), which exists endogenously in pure *Aloe vera* gel [44].

In Figure 2B, there is no change in the position of 1740 cm^−1^ and 1250 cm^−1^ peaks, which corresponds to no change in C=O and C-O-C stretches of carbonyl groups of *O*-acetyl ester. This suggested that the sugar moiety in aloin A and aloin B is D-glucose and the C_1_ position of D-glucose is connected straightforwardly to the C_10_ position of the anthrone ring in a β-configuration. The β-(1–10) C–C bond remains unaffected by acidic and basic conditions [4]. Thus, there is no change in the peaks of anthraquinones in samples exposed to different pHs. The appearance of peaks at 1070 cm^−1^ and 1126 cm^−1^ in all samples (excluding control WLAG)) corresponded to reduction and oxidation to form stable anions [42,43,45]. At acidic pH, the reduction is a single-step two-electron two-proton process, while in alkaline pH the reduction does not involve protons and is only a case of two-electron reduction [46]. This can be verified by HPLC data indicating aloin’s maximum stability at acidic pH (3.5). The appearance of peaks at 1402 cm^−1^ (pH 6.0, pH 6.7) can be assigned to symmetrical -COO stretching of carboxylate, indicating the oxidation of aloin into 10-hydroxyaloin A and B [41].

Femenia and colleagues reported that deacetylation of bioactive components (polysaccharides) and loss of glycosyl residues occurs while drying above 60 °C [47], and according to Minjares-Fuentes et al. mannose units undergo deacetylation (~65%) during industrial drying (spray drying and freeze drying) [24]. As per Figure 2C, peaks at 1250 cm^−1^, 1645 cm^−1^ and 1402 cm^−1^ can be attributed to C-O-C stretches of -COCH_3_ groups: asymmetrical and symmetrical -COO stretching carboxylate compounds, respectively. Thus, the appearance of peaks at 1250 cm^−1^ in FDP and SDP might be due to the loss of galactosyl residues and formation of hydrogen bonds between mannose (sugar) chains, which might be the cause of the loss of aloin, as it was previously reported [48] that acemannan decreased (~40–45%) by the application of hot air (70 °C) and a high-voltage electric field. The peaks at 1402 cm^−1^ can be assigned to symmetrical -COO stretching of carboxylate by oxidation degradation, which intensified after mixing with the organic solvent (methanol) [41], which was not evident in the WLAG. Our results agree with the previous studies of Chokboribal et al. [49]. In the case of FDP and SDP, new band formations at 1070 cm^−1^ and intensified peaks at 1126 cm^−1^ correspond to the glycosidic breakage or liberation of sugars from aloin. However, these bands were absent in WLAG.

### 3.3. Stability of Anthraquinones at Different Temperatures

**Figure 3:**
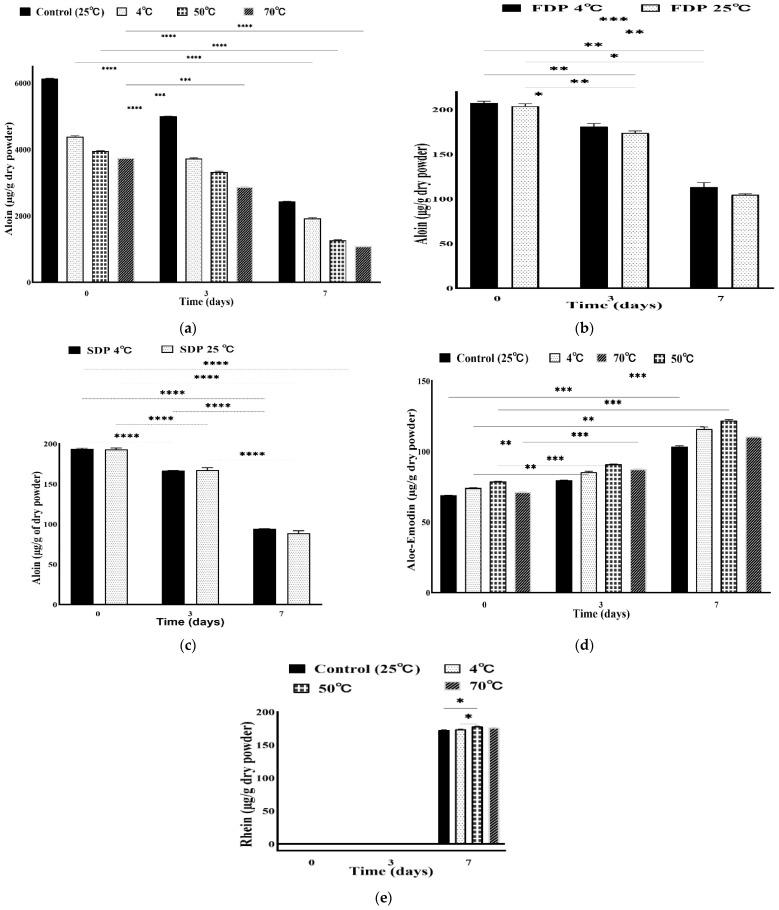
Plots of aloin in whole-leaf *Aloe vera* gel (**a**), aloin in freeze-dried powder of *Aloe vera* (**b**), aloin in spray-dried powder of *Aloe vera* (**c**) and plots of aloe-emodin (**d**) and rhein (**e**) in whole-leaf *Aloe vera* gel, vs. time under various temperature conditions. (Values are from triplicates; the mean and standard deviation are shown. Values significantly different from control to various temperature treatments in a two-way ANOVA with Dunnett’s test analysis indicated with asterisks: * *p*< 0.01, ** *p* < 0.001, *** *p* < 0.0003, **** *p* < 0.0001).

Aloin contents in WLAG were ~6134 µg/g at room temperature (25 °C) and the remaining contents of aloin after various thermal treatments were plotted as per Figure 3a. It is notable from Figure 3a that the aloin degradation occurs gradually while heating WLAG (at 50 °C and 70 °C). WLAG at 70 °C exhibited the highest loss of aloin with an average of 3737.61 µg/g (40% decline) remaining, and 3955.07 µg/g (35% decline) remaining at 50 °C. Aloin A and B are the two diastereoisomers of an anthron C-glycoside, aloin, also referred as barbaloin and isobarbaloin. It has been reported that aloin B (10, C1′:R, S diastereomer of aloin) is formed naturally in plants and the non-enzymatic conversion of aloin B into aloin takes place (C10, C1’:S, S diastereomer of aloin), which might describe their common anthranol form [50]. They differ only in optical rotation and circular dichroism [36]. Therefore, upon heating, aloin A is converted to 10-hydroxyaloins A and B, similar to that previously reported on pure aloin [16]. However, ~4382 µg/g of aloin remained (Figure 3a) at the refrigeration temperature (4 °C) compared to aloin contents (~6134 µg/g) originally present in the control sample at room temperature. The lower amount of aloin at 4 °C might be due to the generation of elgonica dimers A and B, which are major degradation products of aloin A [51].

Figure 3b shows the quantity of aloin in FDP at 4 °C and 25 °C with an average amount of 204.4761 µg/g and 223.15 µg/g, respectively. However, in the case of spray drying, ~193.86 µg/g of aloin at 4 °C and ~220.0 µg/g of aloin at 25 °C were found. Hence, aloin is more stable at room temperature than at refrigeration temperatures in the case of powders as well.

Storage time significantly affected the extent of aloin degradation. It can be elucidated from Figure 3d that degradation of aloin was accelerated while storing at room temperature (from day zero to day three) and boosted from day three to day seven. Decreasing gradually from days (zero to three), the highest loss of aloin was observed on the seventh day in WLAG, FDP and SDP. Remaining aloin contents were 2428.72 µg/g (39%), 113.37 µg/g (50%) and 88.47 µg/g (40%) for WLAG, FDP and SDP, respectively, at room temperature (25 °C). Remarkably, heating samples at 70 °C resulted in ~ 70% reduction of the aloin content. Similar findings were reported previously by Chang et al. [22], that during subsequent heating (60 °C, 70 °C, 80 °C, 90 °C) and storage (12 h) a gradual decrease in aloin contents (also named barbaloin) takes place. However, the similar trend of degradation in FDP was because the freeze-dried powder is very hydrophilic, so there might be a possibility that oxidation caused rapid degradation while exposed to oxygen and extracting solvent [52]. In the case of SDP, the oxidative degradation of aloin into aloe-emodin and various unidentified components occurs, similar to that previously reported on the indispensability of methanol to dissolve barbaloin (aloin) from aloe powder [22]. As per Figure 3a, at 70 °C, more than 60% of aloin degradation was observed on the seventh day, but in powders the degradation was almost 40%.

These findings agreed with the previous study on the instability of barbaloin (also called aloin A), an isomer of aloin, over one month of storage [53]. It was hypothesised that aloin has been converted into its dimers and then trimers during storage. Thermal decomposition of anthrone C-glycoside occurs after heating, resulting in its degradation products including aloe-emodin, a trihydroxyanthraquinone [54]. Figure 3d shows an exciting trend concerning aloe-emodin at various temperatures, showing 69.01 µg/g (25 °C) and 74.15 µg/g of aloe-emodin in the samples heated at 50 °C on day zero. The highest amount in samples at 50 °C vs. 25 °C on day zero is due to the production of aloe-emodin through oxidative degradation of aloin. The aloe-emodin amount on the seventh day was 104.23 02 µg/g, which is almost double as compared to its original amount at 50 °C. This increase is due to metabolite production of aloe-emodin by oxidative cleavage of aloin into its degradation products over time. These results agreed with a previous study [22] that reported the production of aloe-emodin versus the disappearance of aloin over time. In addition, the originally present aloe-emodin in samples was found to be less susceptible to thermal degradation as compared to aloin.

Interestingly, on the seventh day of storage, samples at 25 °C and 70 °C showed a less rapid increase in aloe-emodin contents from their original amount on day zero. The reason is that the disappearance of aloin and the production of aloe-emodin processes are not in equilibrium (more aloin disappeared than in the production of aloe-emodin). Another reason is that racemisation occurs faster at the beginning of degradation. At the start (on day zero), a chemical reaction enhanced aloin’s conversion into aloe-emodin and the thermal decomposition of aloin. However, racemisation was less remarkable than the degradation, until racemic mixtures reached a balance on the third day and finally, racemisation was hardly detected. These findings agree with previous studies [16] that aloin A degradation was proceeded both by degradation and the racemisation mechanism.

Rhein was the last component detected in anthraquinone’s extract from WLAG on the seventh day of storage. In contrast, no peaks of rhein were detected in WLAG on day zero or day three. The highest quantity of rhein detected was 178.25 µg/g at 50 °C, while the lowest was an average of 172.75 µg/g at 25 °C. Apparently, there was no significant difference within all samples. This can be due to the inefficient extraction of rhein with methanol [54]. The production of rhein might be due to the oxidation of aloe-emodin to rhein by a chemical reaction with the organic solvent (methanol). This has been reported previously by [55] and [56], that aloe-emodin is oxidised by chromic acid to rhein. However, no peaks of rhein were observed in FDP or SDP. This might be due to the low content of aloin, which was not enough to be oxidised into aloe-emodin, which produces rhein on further oxidation.

### 3.4. Stability of Anthraquinones at Different pH Adjustments

**Figure 4:**
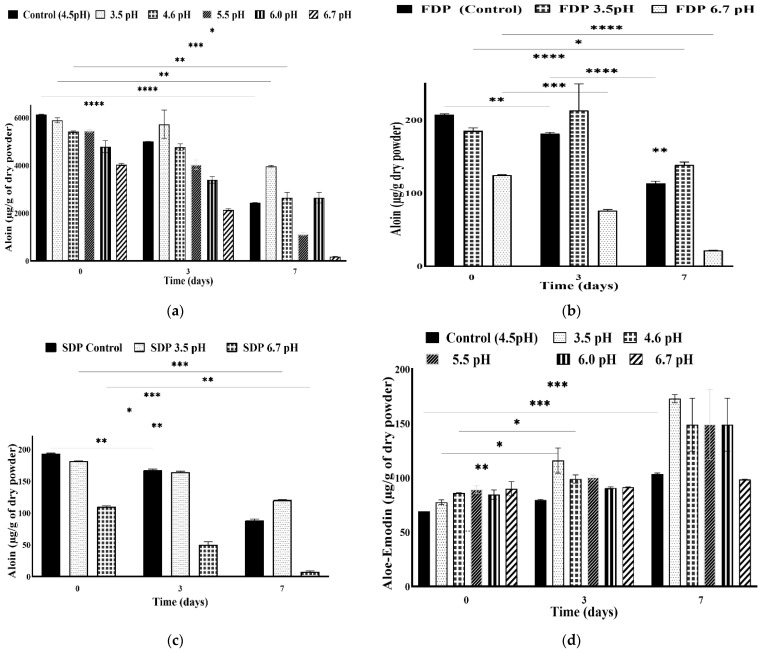

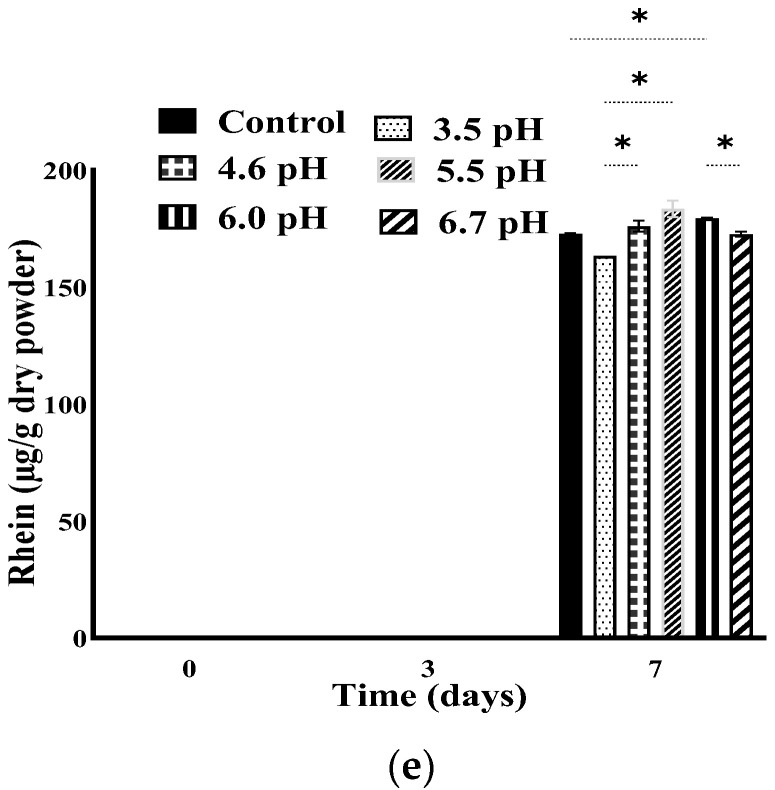
Plots of aloin in whole-leaf *Aloe vera* gel (**a**), aloin in freeze-dried powder of *Aloe vera* (**b**), aloin in spray-dried powder (**c**) of *Aloe vera* and plots of aloe-emodin (**d**) and rhein (**e**) in whole-leaf *Aloe vera* gel, vs. time under various pH conditions. (Values are from triplicates; the mean and standard deviation are shown. Values significantly different from control to various pH treatments in a two-way ANOVA with Dunnett’s test analysis are indicated with asterisks: * *p* < 0.01, ** *p* < 0.001, *** *p* < 0.0003, **** *p* < 0.0001).

Additionally, pH also plays a crucial role in the stability of anthraquinones. A series of pHs (3.5, 4.6, 5.5, 6.0, 6.7) was selected depending on functional foods and nutraceutical requirements. A change in aloin contents in WLAG, FDP and SDP were plotted, as shown in Figure 4a–c, respectively. Aloin contents at natural pH (4.52) of *Aloe vera* gel were ~6134 µg/g, decreasing gradually with an increase in pH from 4.6 (5412 µg/g) to 5.5 (5452 µg/g) and finally to 6.0 (4784.75 µg/g), as shown in Figure 4a. These findings align with [50], who reported that aloin A and B undergo rapid decomposition at basic pH values.

**Figure 5:**
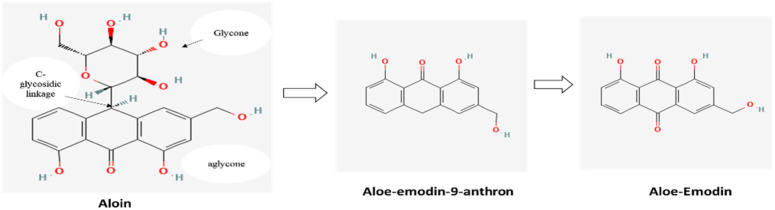
Hydrolysis of aloin into aloe-emodin-9-anthrone and subsequent oxidation to aloe-emodin, a free anthraquinone.

Aloin contents in WLAG exhibited good stability at pH 3.5 with an average amount of 5890.61 µg/g, and lowest stability at pH 6.7 with an average of 4032.70 µg/g compared to the control (6134.0 µg/g) on day zero. Acid hydrolysis might be the reason for the higher amount of aloin at pH 3.5. Sánchez-Viesca and Gómez [57] reported that acid hydrolysis is the starting point of C-glycosidic cleavage, followed by the protonation of O_2_ with the elimination of H_2_O. At a high pH of 6.7, sodium ions combine with carbocation. C-C fission takes place, as well as oxidation into aloe-emodin-nine-anthrone. Then, a second oxido-reduction step necessarily occurs with the formation aloe-emodin, as shown in Figure 5. Finally, the generation of carbocation and ring contraction occurs with oxygen assistance. Therefore, in the presence of hydrochloric acid, a substituted exomethylene derivative must be formed, and no splitting is possible by simple hydrolysis, hence aloin remains stable [58].

Figure 4b shows the quantity of aloin in FDP at pH 3.5 and 6.7 with an average amount of 185.19 µg/g and 124.65 µg/g, respectively, as compared to the control samples (223.15 µg/g). However, in the case of spray drying, 181.45 µg/g of aloin at pH 3.5 and 109.69 µg/g of aloin at pH 6.7 were found, respectively, concerning the control (220.06 µg/g), as depicted in Figure 4c. The identical trend of decreasing the amount of aloin (~18%) at acidic pH in both SDP and FDP was due to the instability of diastereomeric 10-C-glucosylanthrones in solution, where they are supposed to interconvert via tautomeric anthrol [59]. However, in the case of pH 6.7, both powders (FDP, SDP) exhibited parallel aloin reduction (~50%) compared to the control sample. This was due to the oxidation of aloin to its oxanthrone derivatives, 10-hydroxy aloin A and B [60]. By comparing the aloin’s behaviour in all samples (WLAG, FDP, SDP), it can be noted that a less rapid decline (3.9%) can be observed in WLAG as compared to its powders (17%) at acidic pH (3.5). In the aqueous phase in acidic pH (WLAG), the keto form of aloin is more stable than its powders, where the organic solvent favours the enolic form. Therefore, aloin’s stability was higher in WLAG at acidic pH (3.5) than powders.

Regarding storage time (Figure 4a), aloin showed variable trends depending on acidic and basic conditions. WLAG samples (Figure 4a) at natural pH (4.52) with 6134 µg/g of aloin possessed an 18% decrease while moving from days zero to three and a 60% reduction from day zero to day seven. This decline was due to the conversion of aloin to 10 hydroxyaloins A and B while stored at its natural pH (i.e., 4.5). At pH 3.5, aloin exhibited good stability up to day seven, with ~70% of aloin remaining. The stability (90%) from day zero to day three is owed to the keto carbonyl function on the central ring of aloin, which caused aglycones’ elevation after acid hydrolysis. Conversely, the decrease in the aloin contents at acidic pH (20%) was much less than the decrease in aloin from the control WLAG (40%) with seven days of storage. This smaller decline was due to the degradation of aloin into aloe-emodin and elgonica dimers, which occurs quickly at acidic values. These results are aligned with [61], who reported aloin’s inclination after 2 h of acid hydrolysis and then a gradual decrease up to 6 h. In the case of pH 6.7, there was a sharp decline (50%) of aloin from day zero to day three, and less than 2% of aloin was detected on day seven. At alkaline conditions, a more rapid decomposition of aloin into its derivatives (10-hydroxyaloins A and B) took place, as previously reported [16,19].

Regarding Figure 4b,c, FDP and SDP also showed similar trends upon storing. Both powders exhibited good stability of aloin at pH 3.5 for three days, and just 20% degradation was observed up to seven days as compared to control. However, in the case of pH 6.7 samples, 50% of aloin degraded within three days and more than 90% decreased on day seven. Therefore, it was demonstrated that aloin undergoes oxidative degradation at higher pH values and over storage intervals in all samples (WLAG, FDP, SDP). Aloe-emodin was the second anthraquinone component detected in whole-leaf Aloe vera gel with the opposite trend from aloin, and with a slight decline while moving to acidic pH. An average of 69.01 µg/g of aloe-emodin in control WLAG (pH 4.52) and 77.59 µg/g of aloe-emodin at pH 3.5 was present (Figure 4d). However, at pH 4.6, 85.99 µg/g, at pH 5.5, 89.24 µg/g and relatively constant at pH 6.7 with 89.91 µg/g of aloe-emodin were detected. The reason for aloe-emodin concentration being more than the control is due to the production of aloe-emodin at pH 5 or below (one of the degradation products of aloin) that causes the concentration of aloe-emodin to rise while moving from acidic to basic pH (3.5–6.7) values, as previously reported by [19]. Interestingly, at pH 3.5, aloe-emodin contents increased two-thirds from zero to three days and doubled at the end of the seventh day. This was due to the keto-enol tautomerisation of aloe-emodin, whereas, in the case of pH 4.6, 5.5 and 6.0, a slight incline was observed from days zero to three and a 50% increase from its original amount at day zero. This was due to the reason that at higher pH, aloin was converted to aloe-emodin along with other degradation products: 10-hydroxyaloin A and B. Aloe-emodin at pH 6.7 remained steady from day zero to the seventh day due to its stability in basic environment and that there was not sufficient aloin (5% remained at 6.7 pH) that could be converted into its derivative, i.e., aloe-emodin. These results are in alignment with previous studies [19].

Rhein (Figure 4e) was the third anthraquinone found in WLAG samples on the seventh day of storage with an average of 172.75 µg/g. The lowest amount was at pH 3.5 (163.73 µg/g), while the highest amount at (183.50 µg/g) was found at pH 5.5. Apparently, there was no significant difference in all samples. The production of rhein is due to the oxidation of aloe-emodin to rhein by a chemical reaction with an organic solvent. This was previously reported: aloe-emodin is oxidised by chromic acid to rhein [55]. However, no peaks of rhein were observed in freeze-dried and spray-dried powders of whole-leaf *Aloe vera*. This is due to the low contents of aloin, which was not enough to oxidise it into aloe-emodin, which produces rhein on further oxidation.

## 4. Conclusions

The current study indicated that aloin decomposed quickly at higher temperatures (50 °C, 70 °C) and high pH conditions (pH 6.0, 6.7). However, WLAG retains most of its aloin at room temperature and acidic pH. The present study also revealed that aloe-emodin could be retained at high temperatures and at pH 5.0 during processing. However, aloin and aloe-emodin can be converted into degraded products over storage. Therefore, it is recommended that after extraction, the organic solvent must be evaporated to minimise its decomposition and degradation. Rhein was not present in detectable amounts in *Aloe vera* gel. However, on storage for up to seven days, little amounts of rhein were detected due to the degradation of aloe-emodin to rhein. Extra FTIR peaks arose due to the conversion of glycons to aglycon by thermal degradation and oxidation–reduction of anthraquinones. In addition, the extraction efficacy of anthraquinones was more in WLAG than FDP and SDP due to the impact of dehydration stresses on anthraquinones. It is suggested that low temperature and acidic pH are required to preserve aloin contents for functional foods and nutraceuticals to obtain the maximum health benefits.

## Figures and Tables

**Table 1:** Contents of aloin, aloe-emodin and rhein in whole-leaf *Aloe vera* gel (WLAG), freeze-dried powder (FDP) and spray-dried powder (SDP) of *Aloe vera.* (Values are mean and standard deviations from triplicate data. The data were analysed by one-way ANOVA and Tukey’s post hoc HSD tests. The different letters in superscript (a, b, c) within rows indicate statistically significant differences (*p* < 0.05)).

Sample (µg/g of Dry Powder)	Aloin	Aloe-Emodin	Rhein
WLAG	6134.0 ± 15.0 ^a^	69.0 ± 0.0 ^a^	172.7 ± 0.4 ^a^
FDP	223.1 ± 3.9 ^b^	0.00	0.00
SDP	220.0 ± 0.2 ^b^	18.2 ± 0.0 ^c^	0.00

## Data Availability

The data presented in this study are available on request from the corresponding author.
